# Using the mouse to model human disease: increasing validity and reproducibility

**DOI:** 10.1242/dmm.024547

**Published:** 2016-02-01

**Authors:** Monica J. Justice, Paraminder Dhillon

**Affiliations:** 1 Hospital for Sick Children, The Peter Gilgan Centre for Research and Learning, SickKids Research Institute, 686 Bay St, 14.9716, Toronto, Ontario, CanadaM5G 0A4; 2 Disease Models & Mechanisms, The Company of Biologists, Bidder Building, Station Road, Histon, Cambridge CB24 9LF, UK

## Abstract

Experiments that use the mouse as a model for disease have recently come under scrutiny because of the repeated failure of data, particularly derived from preclinical studies, to be replicated or translated to humans. The usefulness of mouse models has been questioned because of irreproducibility and poor recapitulation of human conditions. Newer studies, however, point to bias in reporting results and improper data analysis as key factors that limit reproducibility and validity of preclinical mouse research. Inaccurate and incomplete descriptions of experimental conditions also contribute. Here, we provide guidance on best practice in mouse experimentation, focusing on appropriate selection and validation of the model, sources of variation and their influence on phenotypic outcomes, minimum requirements for control sets, and the importance of rigorous statistics. Our goal is to raise the standards in mouse disease modeling to enhance reproducibility, reliability and clinical translation of findings.

## Abstract (precis)

**Summary:** Raising standards for carrying out and reporting mouse model studies will improve reproducibility and relevance to human disease research.

## Introduction

The mouse is the most commonly used model organism in human disease research (Rosenthal and Brown, 2007). Mouse models have been used extensively to provide insight into the mechanisms underlying many diseases, to explore the efficacy of candidate drugs and to predict patient responses. Despite being such a well-established model, the suitability of the mouse to recapitulate human conditions was called into question by a recent study that compared human and mouse immune responses and reported poor correlation between the two organisms (Seok et al., 2013). The article attracted wide media attention and inspired a flurry of commentaries that debated the validity of the mouse as a disease model. Strikingly, a subsequent paper analyzed the same data, using an arguably more rigorous and less biased methodology, and reported the exact opposite findings, largely restoring faith in the mouse as a model for human conditions (Takao and Miyakawa, 2015).

This high-profile example highlights an issue that has been at the forefront of researchers', publishers' and funding bodies' minds in recent years: the problem of lack of reproducibility in biomedical science. Poor experimental design combined with a lack of rigor in reporting and reviewing has contributed to irreproducibility of findings, which is particularly rife in work that uses preclinical models. In response, the National Institutes of Health (NIH) has called for action to raise standards for carrying out and reporting experiments (Collins and Tabak, 2014). This initiative encourages the scientific community, including funding bodies, academic centers and publishers, to take measures to help enhance reproducibility in science (Kilkenny et al., 2010). As a result, journals including Disease Models & Mechanisms (DMM) recently introduced a compulsory submission checklist that asks authors to verify that they have followed best practice guidelines regarding experimental subjects, data reporting and statistics (http://dmm.biologists.org/sites/default/files/Checklist.pdf).

Below, we discuss some of the common problems that we encounter in manuscripts using mouse models, and provide guidance on avoiding these pitfalls and strengthening papers. By raising awareness and working with authors, we hope to raise the standard of reporting and together combat the issue of irreproducibility.

## Model validity: making sure the mouse is right

It seems an obvious point, but the model used should be appropriate for the question being addressed. An ideal disease model accurately mimics the human condition, genetically, experimentally and/or physiologically. At DMM, we require that the similarities to human disease be rigorously validated, preferably by proof-of-principle experiments demonstrating response to treatment. The controversial study described above compared microarray gene expression data from humans and mice. In one example, data from human blunt-trauma patients were analyzed together with data from a mouse inbred strain that had been exsanguinated. Losing a large amount of blood does not equate to blunt trauma, and so this could be perceived as comparing apples to oranges. Furthermore, inbred mouse strains represent limited genetic diversity and might not reflect the responses generated in a genetically polymorphic human population. The conclusions drawn in this manuscript did not take into account these potential sources of experimental differences between the mouse and human, and raise the possibility of bias in data analysis.

**Figure:**
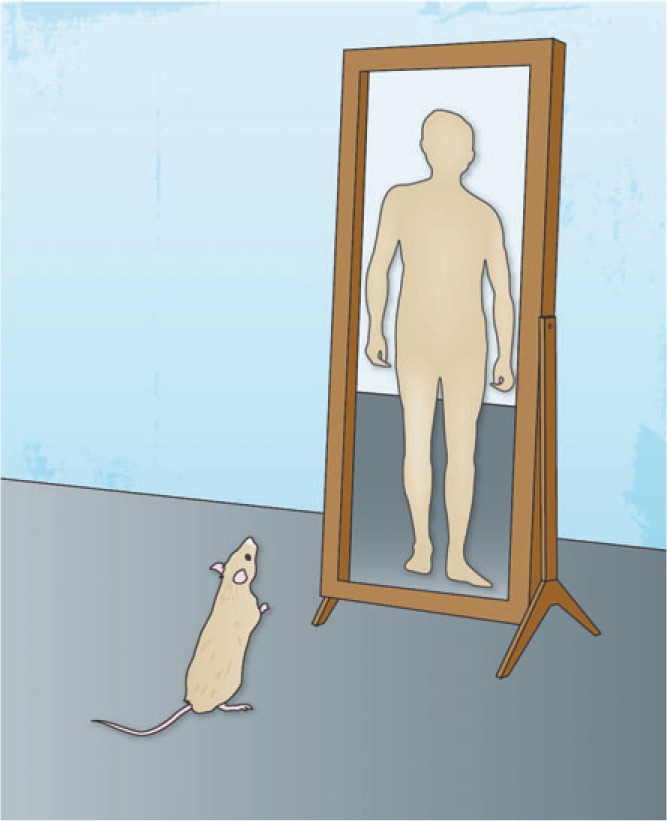

In a different study, a mouse model was reported to display the key motor symptoms seen in humans with amyotrophic lateral sclerosis (ALS) (Wegorzewska et al., 2009). On the basis of this, the model was used in preclinical studies and promising drug candidates were tested in clinical trials; however, these drugs ultimately failed in humans (Perrin, 2014). It was then shown that this mouse is a poor genetic and phenotypic model of the human condition (Hatzipetros et al., 2014). This example illustrates how relevance to the human disease being studied, supported by strong data to validate the use of the model, is crucial for clinical translation. Humanized models – mice expressing human transgenes or engrafted with functional human cells or tissues – can provide important tools to bridge the gap between animals and humans in preclinical research.

## Common sources of variation and how to control for them

As touched upon above, sources of variation that can lead to irreproducible data can be overlooked in mouse papers. The allele and strain in which the target mutation is maintained can make a big difference to the phenotype, so this should be considered during data analysis and a full description of the genetic background should always be included in the Methods section of submitted manuscripts. It should be noted that strains undergo genetic drift when maintained over long periods of time. A key paper reported large molecular differences in 129 substrains maintained in different locations, some of which was due to genetic contamination (Simpson et al., 1997). Similarly, all C57BL/6 strains – commonly used in the laboratory – are not genetically equivalent. The International Mouse Phenotyping Consortium (IMPC) showed that common B6 substrains (C57BL/6J and C57BL/6N) have phenotypic differences (Simon et al., 2013). Therefore, using a homozygous mutant strain that could have been backcrossed to an unknown B6 substrain years ago together with a B6 substrain obtained from a commercial supplier as a control provides a pertinent example of inappropriate use of strains.

The habitat of an animal – determined by the experimental conditions in a laboratory – has a huge impact on experimental results. Husbandry conditions can vary widely, and specific pathogen status, bedding, water, light/dark cycles and group or single housing are just some of the factors that can influence phenotypes. There is mounting evidence to suggest that the bacterial community maintained by an organism, known as the microbiome, can have a strong effect on many aspects of host physiology – from immune responses to neural function (reviewed in Nguyen et al., 2015). Similar to the previous point, mice from the same strain housed in two different locations can have vastly different microbiomes, which could compromise experimental findings. Again, utilizing a control strain from an external supplier, where the experimental conditions could be vastly different, is inappropriate.

Appropriate controls can alleviate misinterpretation of data from such experiments. Littermate controls are ideal for most mouse experiments, and in many experiments can be generated during breeding. However, many strains are maintained as homozygotes over long periods of time, making the use of littermates possible only if the strain is crossed to a relevant B6 substrain, then intercrossed to re-derive the homozygotes. When using mice to test therapeutics, it is also important to randomize the animals to treatment and control groups. Therefore, multiple litters should be used, and littermates should be assigned to each group. Crucially, the experimenter should be blinded to the experimental condition and genotype of the mice, removing any user bias.

## The caveats of conditional mutagenesis

The use of Cre technology together with conditional-ready knockout alleles to eliminate a gene in a specific tissue or at a certain time is a widely used approach. Cre, however, is a recombinase whose expression can have widespread effects on the genome. In 2013, DMM published a paper reporting off-target toxic effects of Cre recombinase in mouse cardiac tissue (Lexow et al., 2013), which called into question the findings from earlier studies that concluded that the phenotypes observed were a consequence of the mutated allele. We thus recommend that, in studies using Cre-based conditional alleles, the effects of Cre activity alone must be included as a control. A companion paper provided evidence that tamoxifen itself can also have harmful effects on the organism (Bersell et al., 2013), which should also be considered when using a tamoxifen-inducible Cre driver. This problem does not stop with Cre. The presence of the tetracycline transactivator (TTA), used to conditionally induce gene expression, can have tissue- and strain-specific effects. For example, its use was reported to cause transgene-specific brain structural anomalies in certain inbred strains of mice (Han et al., 2012). Again, the transgene alone, treated with the inducing agent, is an essential control.

## Statistics and the 3Rs

As in other research areas, rigorous statistics are of critical importance when using mouse models. Mouse studies are regulated by international governing bodies and monitored by institute-specific animal protocol approval committees. The goal in animal studies follows a principle called the ‘3Rs’ – replace, reduce and refine – a policy that provides a framework for humane research (Fenwick et al., 2009). Replacement allows for an alternative to animal research. In the context of DMM, such experiments might provide additional evidence that the animal studies are valid. Refinement should minimize the suffering to the animal and improve animal welfare, a requirement provided by institutional committees prior to experimentation. Reduction minimizes the number of animals used per experiment, which generates a need for careful consideration of statistical methods during experimental design. Because of low numbers, mouse studies use a standard error versus standard deviation as a statistical measure, and such statistics have lower power. To be sure the experiment is valid, studies should use cohort sizes that will provide sufficient statistical power whilst adhering to the 3Rs – studying three or four animals is usually not enough. It is a good idea to consult a statistician in your institute prior to carrying out your experiment, and again when you analyze and report the data.

## From mouse to humans: a community effort

In summary, setting rigorous standards for carrying out and reporting mouse work will help to improve the likelihood of reproducibility. Validation of the model, proper use of controls and attention to rigorous experimentation and statistics are fundamental to increase the translational impact of animal experiments. It is the responsibility of the authors, editors and reviewers to ensure that the common pitfalls described above are avoided and experiments are performed to the highest standard possible within the necessary ethical and regulatory framework.

The debate about the utility of model organisms will never end, and a model is simply that: it is not the human. But there is no denying that the information produced from model organism studies has had a profound and lasting impact on human health, and will continue to do so. An ongoing community effort is needed to promote reproducibility and proper reporting to ensure that the use of preclinical model organisms advances translational research in the most efficient and effective way.
